# Temporal analysis of *Arabidopsis* genes activated by *Eucalyptus grandis* NAC transcription factors associated with xylem fibre and vessel development

**DOI:** 10.1038/s41598-018-29278-w

**Published:** 2018-07-20

**Authors:** M. Laubscher, K. Brown, L. B. Tonfack, A. A. Myburg, E. Mizrachi, S. G. Hussey

**Affiliations:** 1 Department of Biochemistry, Genetics and Microbiology, Forestry and Agricultural Biotechnology Institute (FABI), Genomics Research Institute (GRI), University of Pretoria, Private Bag X28, Pretoria, 0002 South Africa; 2 Plant Physiology and Improvement Unit, Laboratory of Biotechnology and Environment, Department of Plant Biology, University of Yaoundé I, P.O. Box 812, Yaoundé, Cameroon

## Abstract

Secondary cell wall (SCW) deposition in *Arabidopsis* is regulated among others by NAC transcription factors, where SND1 chiefly initiates xylem fibre differentiation while VND6 controls metaxylem vessel SCW development, especially programmed cell death and wall patterning. The translational relevance of *Arabidopsis* SCW regulation theory and the utility of characterized transcription factors as modular synthetic biology tools for improving commercial fibre crops is unclear. We investigated inter-lineage gene activation dynamics for potential fibre and vessel differentiation regulators from the widely grown hardwood *Eucalyptus grandis* (Myrtales). *EgrNAC26*, a *VND6* homolog, and *EgrNAC61*, an *SND1* homolog, were transiently expressed in *Arabidopsis* mesophyll protoplasts in parallel to determine early and late (i.e. 7 and 14 hours post-transfection) gene targets. Surprisingly, across the time series EgrNAC26 activated only a subset of SCW-related transcription factors and biosynthetic genes activated by EgrNAC61, specializing instead in targeting vessel-specific wall pit and programmed cell death markers. Promoters of *EgrNAC26* and *EgrNAC61* both induced reporter gene expression in vessels of young *Arabidopsis* plants, with *EgrNAC61* also conferring xylem- and cork cambium-preferential expression in *Populus*. Our results demonstrate partial conservation, with notable exceptions, of SND1 and VND6 homologs in *Eucalyptus* and a first report of cork cambium expression for EgrNAC61.

## Introduction

Secondary cell walls (SCWs) equip plants with pathogen resistance, mechanical support and the ability to effectively transport water from the roots to the aerial organs^1–3^. Fast-growing angiosperm trees such as *Eucalyptus* are widely grown as short-rotation lignocellulosic feedstocks for pulp, paper and other renewable biomass products derived from the SCWs within wood^4^. The deposition of SCWs, which consist primarily of cellulose, hemicelluloses and lignin, is regulated by a complex semi-hierarchical transcriptional network composed mainly of NAC (NAM/ATAF/CUC) and MYELOBLASTOSIS (MYB) transcription factors (TFs)^5–8^. Among the known master regulators of SCW formation in the herbaceous model *Arabidopsis thaliana* (Arabidopsis), Secondary Wall NACs (SWNs^9^) appear to initiate SCW deposition through this regulatory network, occupying the upper network tier and regulating several mid- and lower-tier TFs as well as core SCW biosynthesis genes. The SWNs directly regulate, among others, the expression of several key MYB TF genes, with MYB46 and its functionally redundant homolog MYB83 also being considered master regulators positioned mid-tier, as is the CCCH-type zinc finger C3H14^10–14^.

Arabidopsis SWNs in the NST clade, among them NST1 (NAC SECONDARY WALL THICKENING PROMOTING FACTOR 1), NST2 and SND1 (SECONDARY WALL-ASSOCIATED NAC DOMAIN THICKENING FACTOR 1), regulate SCW formation in fibres, anther endothecia and silique valves to a large degree of redundancy, while VND (VASCULAR-RELATED NAC DOMAIN) clade TFs encompassing VND1 through VND7 are vessel-specific, with VND6 specifically regulating metaxylem SCW deposition and VND7 regulating both meta- and protoxylem vessel formation^15–24^. Thus, VND6 is a key regulator of the reticulated and pitted wall patterning observed in secondary xylem vessels, the deposition of which is determined by the bundled microtubule structure of the cytoskeleton^25,26^. In woody angiosperms such as *Populus* the expression of NST and VND clade homologs appear to overlap somewhat, with both the *SND1* homolog *PtrWND1B* (a *Populus trichocarpa* wood-associated NAC domain protein) and the *VND7* homolog *PtrWND6A* being expressed in xylem and phloem fibres, while vessel-specific differentiation appears to be regulated by the exclusive expression of *PtrWND6A* in vessels^27,28^. High-resolution spatial transcript profiling in aspen from phloem through the cambium to the lignified xylem zone revealed biphasic expression peaks for *SND1* homologs in phloem and early xylem SCW deposition, while *VND6* homolog transcripts peaked either during xylem SCW deposition or its cessation^29^, suggesting a specialization of *SND1* homologs in phloem and xylem fibre formation and *VND6* homologs in vessel differentiation. However, dominant repression in *Populus* of either the SND1 homolog PtrWND2B or the VND7 homolog PtrWND6B resulted in significantly reduced xylem SCW deposition in both fibres and vessels^30^. Although this suggests less distinct roles for SWN-mediated regulation of fibre and vessel SCW formation in woody angiosperms, the question of partially overlapping versus distinct roles in secondary xylem development remains poorly resolved. For example, homologs of *SND1* and *VND6*/*VND7* in monocots (a lineage lacking secondary xylem derived from a vascular cambium) appear to be expressed indistinguishably in sclerenchyma fibres and vessels^31,32^, while in Norway Spruce (a woody gymnosperm lacking fibres and vessels) sufficiently distinct roles could be inferred for VND and NST homologs during xylogenesis^33^.

Xylem fibre and vessel differentiation is distinguished by differences in SCW thickness and patterning as well as the timing and rate of programmed cell death (PCD) and autolysis, which in the case of water-conducting vessel elements yields hollow lumens shortly after SCW deposition^6,34,35^. Congruent with their proposed functions in xylem vessel development, VND6 and VND7 strongly activate PCD genes in Arabidopsis^9,36^. PCD and autolysis, which are distinct biological processes, are initiated by Ca^2+^ influx signals resulting from extracellular proteolysis by serine proteases^37,38^. Proteins currently known to be involved in vessel autolysis include XYLEM CYSTEINE PEPTIDASE 1 (XCP1), XCP2 and METACASPASE 9 (MC9) that together cause autolysis of the cytoplasm, the endonuclease ZEN1, XYLEM SERINE PEPTIDASE 1 (XSP1), and possibly ARABIDOPSIS THALIANA SUBTILASE 1.1 (SBT1.1)^39–44^. The later onset of PCD in fibres may be attributed to the weaker activation of PCD genes by SND1 and slower accumulation of the necessary enzymes to prompt cellular degradation^45,46^. Compared to fibres, metaxylem vessels exhibit extensive, pronounced pits in their SCWs that allow for the lateral movement of water to living fibres and xylem parenchyma^47,48^. Pits are established by the recruitment of microtubule disassembly proteins MIDD1 and Kinesin 13 A by pit localization of GTP-bound ROP11, a Rho GTPase that is localized to oval planar domains at the plasma membrane by IQD13, activated by ROPGEF4 and deactivated by ROPGAP3^49–51^.

Despite what is believed to be substantial evolutionary conservation in SCW transcriptional regulation^52^, the prevalence and significance of lineage-specific adaptations to the SCW transcriptional network architecture among angiosperms are poorly understood. In *Populus*, for example, numerous co-orthologs of *SND1* exist, of which alternative splicing of *PtrWND1B* in particular produces full-length and intron-retaining, truncated isoforms of the protein that can form non-functional heterodimers^27,53–55^. Since Arabidopsis does not harbour known *SND1* splice variants, this shows that *SND1*-mediated SCW regulation may differ between plant lineages. In the *Eucalyptus* woody model, a small number of studies have aided in our understanding of SCW regulation^56–61^. To date, our knowledge of the functions of SWN master regulators in *Eucalyptus* is limited to a few gene targets of EgWND1, an *E*. *gunnii* homolog of SND1^30^, with some evidence that an intron 2-retaining splice variant may also exist in *E*. *grandis*^27^. In previous work^62^, we identified putative *E*. *grandis* orthologs of SND1 (EgrNAC61, also orthologous to EgWND1) and VND6 (EgrNAC26) which we hypothesised regulate fibre and vessel SCW development, respectively.

While functionally characterized SCW TFs and their promoters are potential tools for the re-engineering of woody biomass, their crude manipulation generally results in undesirable phenotypes and hence refined approaches adopting synthetic and systems biology are favourable^63^. The development of high-precision multipartite synthetic gene constructs, however, assumes individual parts possess modularity (functional fidelity of discrete parts in different host organisms) and orthogonality (independence from and minimal interference by native endogenous processes), both of which are poorly demonstrated for SCW TFs. For instance, what gene targets might we expect to be activated when SCW TF homologs from diverged lineages are heterologously and ectopically induced, and how is this influenced when their endogenous promoters are exchanged with synthetic promoters? In this study, we functionally characterized *E*. *grandis* SCW master regulators through observation of their SCW network targets over time in the context of Arabidopsis mesophyll protoplasts, and their promoter activities in woody and non-woody models. Our results show that EgrNAC61 is a more potent activator of SCW genes while the VND6 homolog EgrNAC26 mainly regulates PCD and autolysis in vessels.

## Results

### *EgrNAC26* and *EgrNAC61* transcript splicing, transcriptional activity and spatial expression patterns

**Figure 1:**
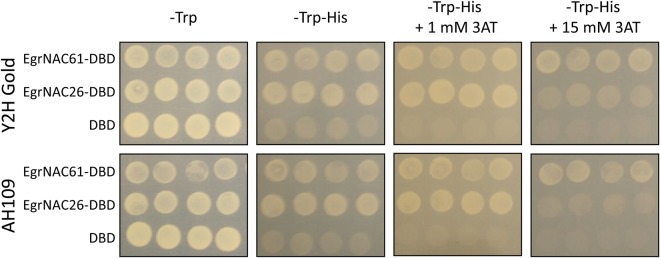
Transactivation of the *HIS* reporter gene in two yeast strains (Y2H Gold and AH109) by EgrNAC61 and EgrNAC26 fused to the GAL4 DNA binding domain (DBD). Yeast transformed with all constructs, including empty vector encoding only the DBD, grew on media lacking Tryptophan (-Trp). Growth of EgrNAC61-DBD and EgrNAC26-DBD fusions on media lacking Tryptophan and Histidine (-Trp-His) demonstrate transcriptional transactivation, while growth on increasing concentrations of 3AT indicate the strength of transcriptional transactivation.

We PCR-amplified the *EgrNAC26* and *EgrNAC61* coding sequences from *E*. *grandis* developing secondary xylem cDNA for cloning. We found no evidence of alternative splice variants among the amplicons (Fig. S1). Similarly, analysis of RNA-seq reads from an independent developing secondary xylem sample^64,65^ showed no significant coverage across any of the introns (Fig. S2). These results therefore do not support those of the previously observed *EgrNAC61* intron 2 retention reported by Zhao *et al*.^27^. Since EgrNAC26 and EgrNAC61 are likely master regulators of SCW deposition in *E*. *grandis* based on homology, we next tested the ability of these proteins to activate transcription of the *His* reporter gene in two strains of *S*. *cerevisiae*. Both strains expressing either EgrNAC61 or EgrNAC26 grew on histidine-deficient media supplemented with up to 15 mM of the *His* reporter inhibitor 3AT (Fig. 1), while for the empty-vector control line significantly less growth was observed on media lacking histidine and no growth was observed on media supplemented with 3AT. For both strains, stronger reporter gene activation at 15 mM 3AT was observed for EgrNAC61 than EgrNAC26. These results demonstrate that EgrNAC26 and EgrNAC61 have strong intrinsic transcriptional activation activities.

**Figure 2:**
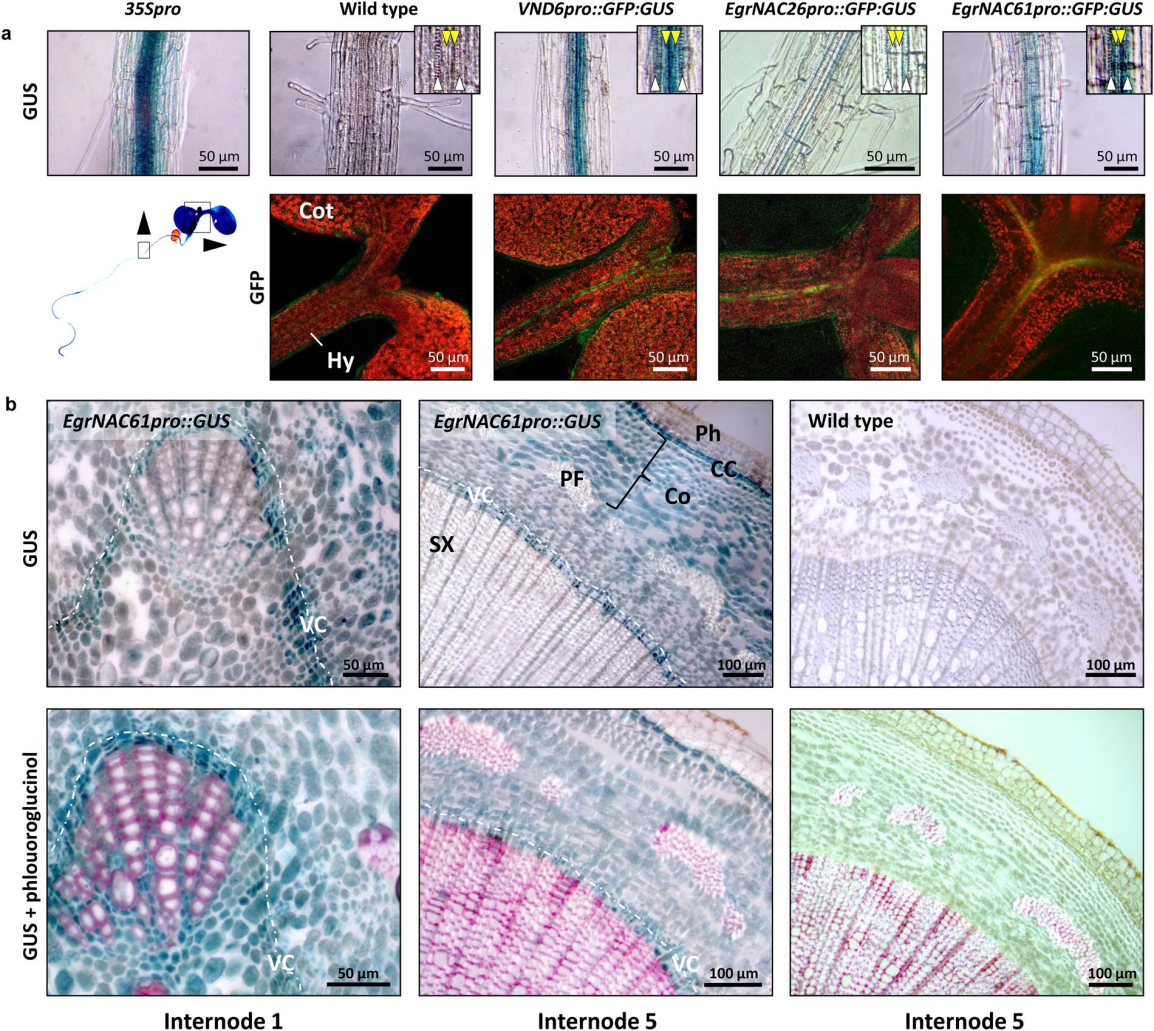
Reporter gene analysis of *EgrNAC26* and *EgrNAC61* promoters. (**a**) Promoter analysis in Arabidopsis seedlings. Top panel, GUS histochemical staining in seedling roots. Insets show the positions of protoxylem (white chevrons) and metaxylem (yellow chevrons) vessels. *35* *Spro*, CaMV *35S* promoter (positive control). Bottom panel, corresponding GFP confocal microscopy analysis of seedling shoot tips for each construct. Cot, cotyledon; Hy, hypocotyl. (**b**) *EgrNAC61 promoter::GUS* analysis of internodes 1 and 5 in transgenic poplar plants. Bottom panel, phloroglucinol-stained GUS sections. CC, cork cambium; Co, cortex; PF, phloem fibres; Ph, phellem; SX, secondary xylem; VC, vascular cambium.

We analysed *promoter::GFP:GUS* reporter fusions of the *EgrNAC26 and EgrNAC61* promoter sequences to determine their heterologous expression patterns. In the vasculature of Arabidopsis seedling roots, which contain only xylem vessels, the metaxylem vessel marker *VND6pro::GFP:GUS* produced predominant GUS staining in the metaxylem vessels in the centre of the vasculature, with less blue staining in protoxylem vessels (Fig. 2a). In contrast, a 35*Spro::GFP:GUS* constitutive control showed staining in all root cell types. In most cases, GUS staining in *EgrNAC26pro::GFP:GUS* lines was not visible; in a few cases, faint expression was observed in the protoxylem vessels. However, GFP expression for this construct was clearly detected in the seedling hypocotyl vasculature (Fig. 2a). Previously, *SND1* promoter-directed expression was observed predominantly in interfascicular and xylary fibres of inflorescence stems and hypocotyls of flowering Arabidopsis plants, and also in developing vessels undergoing SCW thickening^16,20^. In *EgrNAC61pro::GFP:GUS* plants, GUS staining was evident in proto- and metaxylem vessels in the root vasculature (Fig. 2a). Analysis of GFP expression in the vasculature of seedling hypocotyls for the *VND6*, *EgrNAC26* and *EgrNAC61* promoters corroborates our findings of GUS staining in the seedling vessels (Fig. 2a).

We also explored the expression patterns of *EgrNAC61* during wood formation through *promoter::GUS* analysis in transgenic poplar plants. GUS signal was strongest in the developing xylem region of the first internode, just before lignification of the primary xylem vessels distinguishable with phloroglucinol staining (Fig. 2b). In secondary xylem in the fifth internode, expression was observed throughout the cortex but was strongest in the xylem expansion zone, sandwiched by the vascular cambium and the lignification zone, as well as in the cork cambial region (phellogen). The latter is a novel report. GUS expression appeared to be visible in developing secondary vessel and fibre cells, forming a near-continuous ring of signal along the expansion zone (Fig. 2b, internode 5). Together, the Arabidopsis and poplar results support a role for EgrNAC26 in the regulation of vessel formation, and EgrNAC61 in the regulation of secondary xylem fibre and vessel deposition.

### Identification of early and late transcriptional targets of EgrNAC26 and EgrNAC61 in Arabidopsis protoplasts

In order to identify early (likely direct) and late (likely indirect) gene targets of EgrNAC26 and EgrNAC61 in a heterologous background, we transiently overexpressed these genes in linkage with a dual GFP reporter cassette in Arabidopsis leaf mesophyll protoplasts. Total RNA was isolated 7 and 14 hours post-transfection (hpt) from three independent protoplast isolations per time point. Transfection efficiencies were approximately 30–50% (Fig. S3). Following RNA quality analysis (Fig. S4) and strand-specific RNA sequencing, between 17 and 36 million paired-end reads were obtained per library, most with >90% concordant mapping of read pairs to the Arabidopsis TAIR10 genome (Table S2). After quantification and normalization of the expression values, all transgene FPKM values, including those of *GFP* for the empty vector (EV) libraries, were within the top percentile of the detected transcript levels and expressed at similar levels across time points and replicates (Fig. S5). Principle Component Analysis (PCA) of transcriptome-wide FPKM values for each library showed stronger differentiation according to time point than construct, while clear clustering by construct was only apparent at 7 hpt (Fig. S6), suggesting that each master regulator affected only a small proportion of the transcriptome uniquely, and most distinguishably for the early targets.

**Figure 3:**
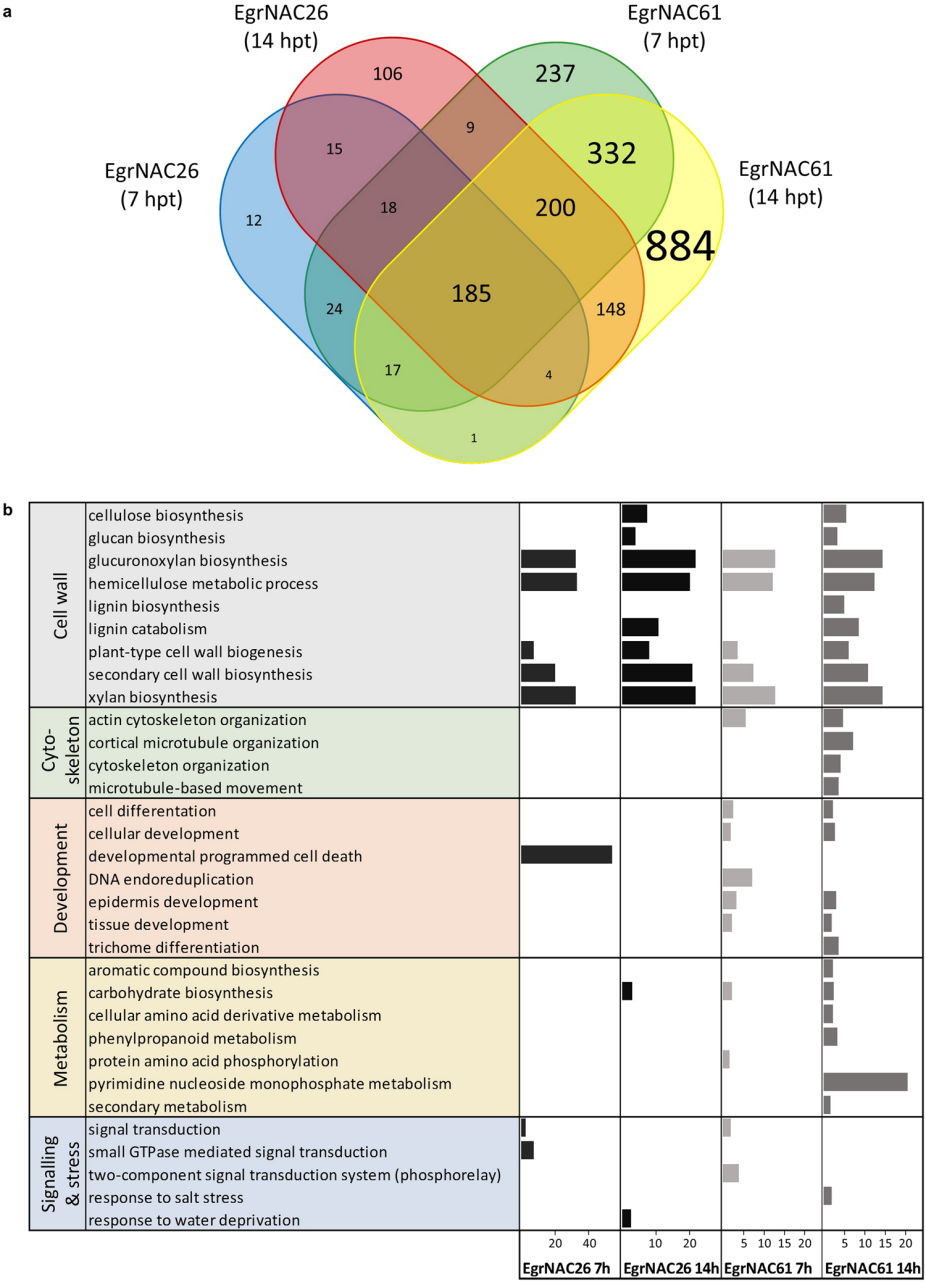
Differentially expressed genes (DEGs) induced by EgrNAC26 and EgrNAC61. (**a**) Common and unique DEGs for EgrNAC26-OX and EgrNAC61-OX datasets at 7 and 14 hpt with font sizes scaled to the number of genes in each cell. (**b**) Selected overrepresented biological functions among EgrNAC26-OX and EgrNAC61-OX genes, with fold enrichment indicated on the x-axis.

Differential expression analysis of the mesophyll protoplasts induced with *EgrNAC26* (EgrNAC26-OX) and *EgrNAC61* (EgrNAC61-OX) followed by FPKM and fold-change filtering (FPKM ≥1, fold-change ≥2-fold up or down) identified 276 (EgrNAC26; 7 hpt), 1,022 (EgrNAC61; 7 hpt), 685 (EgrNAC26; 14 hpt) and 1,771 (EgrNAC61; 14 hpt) differentially expressed genes (DEGs), of which the vast majority were upregulated (Figs 3a, S7, Supplementary Dataset 1). The overlap between EgrNAC26-OX and EgrNAC61-OX datasets was highly significant (*P* < 2.2 × 10^−16^), only one transcript was alternately up- or down-regulated across time points, and DEGs common to EgrNAC26-OX and EgrNAC61-OX datasets were all up- or down-regulated concordantly, demonstrating robust and consistent activation of a subset of shared genes. Overrepresented biological processes were identified for EgrNAC26-OX and EgrNAC61-OX DEGs through Gene Ontology (GO) enrichment analysis. Significantly overrepresented terms among up-regulated DEGs in the EgrNAC26*-*OX and EgrNAC61-OX datasets were largely associated with SCW biosynthesis, particularly at 14 hpt. Common terms included secondary cell wall biogenesis, cellulose biosynthesis and (glucurono)xylan biosynthesis (Fig. 3b), supporting the hypothesized roles of these genes as SCW master regulators since the overexpression of the transgenes was sufficient to cause ectopic activation of these genes. Differences between the datasets provided insight into known and novel biological functions that were likely distinctly regulated by EgrNAC26 and EgrNAC61. Specifically, supporting evidence for the role of EgrNAC26 as a vessel-preferential master regulator was the enrichment of genes in the category “developmental PCD” at 7 hpt (Fig. 3b). In contrast, EgrNAC61 induced a good representation of cytoskeleton-related processes, a number of developmental processes such as trichome and epidermal differentiation, and interestingly several primary metabolic processes such as amino acid and pyrimidine metabolism in addition to secondary metabolism. These results are consistent with a role of *EgrNAC26* and *EgrNAC61* in the regulation of SCW synthesis.

### Temporal regulation of Arabidopsis SCW transcriptional network targets by EgrNAC26 and EgrNAC61

Notable DEGs in the EgrNAC26-OX and EgrNAC61-OX datasets involved in SCW xylem development and transcriptional regulation are shown in Table 1. In total, 151 TFs were differentially expressed, most of them upregulated (Table S3). Many genes were exclusively up-regulated in EgrNAC61-OX protoplasts, among them known SCW transcriptional regulators *MYB4*, *MYB7*, *MYB42*, *MYB52*, *MYB54*, *MYB83* and *MYB103*, and lignin regulators *MYB58*, *MYB63* and *MYB85*^11,13,66,67^ (Tables 1, S3). Likewise, several lignin-specific genes were upregulated in EgrNAC61-OX at 14 hpt only, including *4CL3*, *CCoAOMT1*, and *HCT*. Interestingly, far fewer SCW-related DEGs were unique to EgrNAC26-OX; noteworthy examples included *LBD30*/*ASL19*, *VND2*, *TED7*, a key cytoskeleton-associated protein *MAP70-5* and the ROP11 activator *ROPGEF4* (Table 1). No activation of the endogenous *SND1* gene was observed, suggesting that the self-activation of SND1 observed in plasmid-based promoter transactivation and electrophoretic mobility shift assays^68^ did occur in the context of native mesophyll chromatin.

**Table 1:** Secondary cell wall-associated genes regulated by EgrNAC26 and EgrNAC61.

Gene ID	Gene name	Linear fold change			
		EgrNAC26-OX		EgrNAC61-OX	
		7 hpt	14 hpt	7 hpt	14 hpt
**Transcription factors**					
AT1G75410	BEL1-LIKE HOMEODOMAIN 3 (BLH3)	—	—	3.4	4.4
AT4G34610	BEL1-LIKE HOMEODOMAIN 6 (BLH6)	—	18.5	12.9	361.7
AT1G19700	BEL1-LIKE HOMEODOMAIN 10 (BEL10)	—	—	3.5	5.7
AT1G66810	CCCH ZINC FINGER 14 (C3H14)	—	5.2	7.5	34.0
AT1G62990	KNOTTED-LIKE HOMEOBOX OF ARABIDOPSIS THALIANA 7 (KNAT7)	—	162.4	108.8	1139.7
AT2G40470	LATERAL ORGAN BOUNDARIES 15 (BD15)/ASL11	—	26.4	—	49.2
AT4G00220	LATERAL ORGAN BOUNDARIES 30 (LBD30)/ASL19	—	Inf*	—	—
AT1G66230	MYB20	2.2	—	3.8	2.4
AT4G12350	MYB42	—	—	—	3.5
AT5G12870	MYB46	—	255.9	—	1161.7
AT1G17950	MYB52	—	—	—	Inf*
AT1G73410	MYB54	—	—	—	84.8
AT4G01680	MYB55	9.3	3.8	22.6	4.9
AT1G16490	MYB58	—	—	—	28.3
AT1G79180	MYB63	—	—	—	5.2
AT3G08500	MYB83	—	—	Inf*	Inf*
AT4G22680	MYB85	—	—	6.9	6.8
AT1G63910	MYB103	—	—	Inf*	—
AT2G38090	MYB-R	—	12.3	3.5	48.4
AT2G34710	PHABULOSA (PHB) / ATHB14	—	—	2.2	2.6
AT4G28500	SECONDARY WALL-ASSOCIATED NAC DOMAIN 2 (SND2)	—	42.4	—	1085.8
AT1G28470	SECONDARY WALL-ASSOCIATED NAC DOMAIN (SND3)	—	88.1	33.4	416.9
AT5G60200	TARGET OF MONOPTEROS 6 (TMO6)	—	—	2.8	3.4
AT4G36160	VASCULAR-RELATED NAC-DOMAIN 2 (VND2)	—	2.1	—	—
AT5G64530	XYLEM NAC DOMAIN 1 (XND1)	—	—	2.5	—
**Cell wall biosynthesis**					
AT1G65060	4-COUMARATE:CoA LIGASE 3 (4CL3)	—	—	—	61.1
AT4G34050	CAFFEOYL-CoA 3-O-METHYLTRANSFERASE 1 (CCoAOMT1)	—	—	—	2.9
AT4G26220	CAFFEOYL CoA ESTER O-METHYLTRANSFERASE 7 (CCoAOMT7)	—	9.7	—	271.3
AT4G39350	CELLULOSE SYNTHASE 2 (CESA2)	—	—	—	2.1
AT5G44030	CELLULOSE SYNTHASE 4 (CESA4)	—	35.3	29.7	649.5
AT5G09870	CELLULOSE SYNTHASE 5 (CESA5)	—	—	3.1	5.1
AT5G17420	CELLULOSE SYNTHASE 7 (CESA7)	—	49.9	42.0	728.2
AT4G18780	CELLULOSE SYNTHASE 8 (CESA8)	—	91.3	—	1792.6
AT5G03760	CELLULOSE SYNTHASE-LIKE A9 (CSLA09)	—	8.1	—	45.1
AT2G32620	CELLULOSE SYNTHASE-LIKE B2 (CSLB02)	—	56.2	Inf*	1331.2
AT1G15950	CINNAMOYL COA REDUCTASE 1 (CCR1)/IRREGULAR XYLEM 4 (IRX4)	—	—	—	2.1
AT5G15630	COBRA-LIKE4 (COBL4)	—	44.1	—	1083.1
AT2G40890	COUMARATE 3-HYDROXYLASE 1 (C3H1)/ REF8	—	—	—	2.2
AT3G55990	ESKIMO 1 (ESK1)	—	220.5	—	1676.0
AT5G47820	FRAGILE FIBER1 (FRA1)	—	—	2.2	2.6
AT2G28110	FRAGILE FIBER8 (FRA8)/IRREGULAR XYLEM7 (IRX7)	2.3	3.2	6.1	10.6
AT3G18660	GLUCURONIC ACID SUBSTITUTION OF XYLAN 1 (GUX1)	—	411.2	—	2763.5
AT4G33330	GLUCURONIC ACID SUBSTITUTION OF XYLAN 2 (GUX2)	—	167.0	Inf*	1922.8
AT5G48930	HYDROXYCINNAMOYL-CoA SHIKIMATE/QUINATE HYDROXYCINNAMOYL TRANSFERASE (HCT)	—	—	—	2.3
AT2G37090	IRREGULAR XYLEM 9 (IRX9)	—	103.5	—	2449.4
AT1G27440	IRREGULAR XYLEM 10 (IRX10)	3.2	7.1	11.5	107.4
AT5G61840	IRX10-LIKE (IRX10-L)	—	—	—	4.1
AT4G36890	IRREGULAR XYLEM 14 (IRX14)	—	—	2.1	7.6
AT5G67230	IRX14-LIKE (IRX14-L)	—	8.1	—	62.3
AT3G50220	IRREGULAR XYLEM 15 (IRX15)	—	173.9	—	1575.3
AT5G67210	IRX15-LIKE (IRX15-L)	—	12.9	—	109.1
AT2G38080	LACCASE 4 (LAC4)	19.2	206.1	71.2	1419.8
AT5G60020	LACCASE 17 (LAC17)	—	110.9	—	423.5
AT1G19300	PARVUS	3.0	38.6	22.5	268.9
AT3G47400	Pectinesterase family protein	Inf*	555.9	Inf*	889.7
AT5G51890	PEROXIDASE 66 (PER66)	—	139.6	132.1	1690.6
AT5G66390	PEROXIDASE 72 (PER72)	—	3564.3	—	149.3
AT1G70500	Polygalacturonase	466.9	546.8	538.8	608.7
AT1G23460	Polygalacturonase	3.6	22.8	3.8	18.1
AT2G43880	Polygalacturonase, putative / pectinase, putative	—	—	—	Inf*
AT5G46340	REDUCED WALL ACETYLATION 1 (RWA1)	—	2.8	7.8	33.9
AT2G34410	REDUCED WALL ACETYLATION 3 (RWA3)	—	—	3.6	12.5
AT1G29890	REDUCED WALL ACETYLATION 4 (RWA4)	—	2.6	4.1	15.1
AT5G01360	TRICHOME BIREFRINGENCE-LIKE 31 (TBL31)	—	112.2	—	562.8
AT2G40320	TRICHOME BIREFRINGENCE-LIKE 33 (TBL33)	—	125.0	Inf*	2067.0
AT2G38320	TRICHOME BIREFRINGENCE-LIKE 34 (TBL34)	—	761.1	—	1268.2
AT1G12780	UDP-GLC 4-EPIMERASE 1 (UGE1)	2.6	2.7	8.2	8.6
AT5G59290	UDP-XYLOSE SYNTHASE 3 (UXS3)	2.0	7.4	10.4	60.4
AT2G28760	UDP-XYLOSE SYNTHASE 6 (UXS6)	9.9	40.8	60.1	242.3
AT2G14620	XYLOGLUCAN ENDOTRANSGLYCOSYLASE/HYDROLASE 10 (XTH10)	6.0	8.0	36.1	9.4
AT3G48580	XYLOGLUCAN ENDOTRANSGLYCOSYLASE/HYDROLASE 11 (XTH11)	—	3.0	2.4	2.8
**Cytoskeleton**					
AT1G14840	MICROTUBULE-ASSOCIATED PROTEIN 70-5 (MAP70-5)	—	63.4	—	—
AT3G53350	MICROTUBULE DEPLETION DOMAIN 1 (MIDD1)	—	2.6	5.7	11.3
AT1G50010	TUBULIN ALPHA-2 CHAIN (TUA2)	—	5.1	4.3	21.4
AT1G04820	TUBULIN ALPHA-4 CHAIN (TUA4)	—	—	—	3.6
AT4G14960	TUBULIN ALPHA-6 CHAIN (TUA6)	—	—	—	3.8
AT5G23860	TUBULIN BETA-8 (TUB8)	2.3	3.6	6.3	19.8
AT3G16630	Kinesin-13A	—	—	—	2.5
**Signaling**					
AT4G13195	CLE44	—	—	0.4	0.3
AT4G17220	IQ-DOMAIN 10 (IQD10)	—	—	—	Inf*
AT2G45890	RHO GUANYL-NUCLEOTIDE EXCHANGE FACTOR 4 (ROPGEF4)	—	39.2	—	—
AT2G46710	RHO GTPASE-ACTIVATING PROTEIN 3 (ROPGAP3)	2.7	6.4	29.7	59.0
AT5G62880	RHO-RELATED PROTEIN FROM PLANTS 11 (ROP11)	2.7	3.3	6.2	7.2
AT1G08340	RAC GTPase	—	55.7	—	1020.4
**Programmed cell death and autolysis**					
AT1G11190	BIFUNCTIONAL NUCLEASE 1 (BFN1)	—	359.3	—	147.9
AT5G04200	METACASPASE 9 (MC9)	90.3	112.3	87.1	44.5
AT1G26820	RIBONUCLEASE 3 (RNS3)	Inf*	678.7	Inf*	926.7
AT4G35350	XYLEM CYSTEINE PEPTIDASE 1 (XCP1)	423.0	1371.1	117.7	119.9
AT1G20850	XYLEM CYSTEINE PEPTIDASE 2 (XCP2)	83.9	1208.3	45.0	126.4
**Miscellaneous genes linked to xylem development**					
AT3G16920	CHITINASE-LIKE PROTEIN 2 (CTL2)	6.7	62.6	96.0	890.8
AT3G62020	Germin-like protein 10 (GLP10)	4.2	48.1	16.0	348.2
AT1G33800	Glucuronoxylan(GX)-specific 4-O-methyltransferase	—	97.3	167.6	1165.0
AT1G31720	MODIFYING WALL LIGNIN 1 (MWL1)	—	Inf*	—	Inf*
AT4G27435	MODIFYING WALL LIGNIN 2 (MWL2)	—	68.3	—	1626.9
AT5G18460	Protein of Unknown Function (DUF239)	242.1	295.1	651.0	453.4
AT4G09990	Protein of Unknown Function (DUF579)	—	8.6	8.1	113.8
AT4G18425	Protein of Unknown Function (DUF679)	Inf*	—	Inf*	—
AT1G43790	TRACHEARY ELEMENT DIFFERENTIATION-RELATED 6 (TED6)	—	432.0	—	624.6
AT5G48920	TRACHEARY ELEMENT DIFFERENTIATION-RELATED 7 (TED7)	18.2	—	—	—

*Inf refers to instances where the FPKM was 0 in the empty-vector control, but was larger than 1 in the test sample.

hpt, hours post transfection.

Several SCW-related genes and TFs were upregulated in at least one time point for both EgrNAC26-OX and EgrNAC61-OX. However, we noted that apart from the abovementioned SCW-related genes and TFs being exclusively activated by EgrNAC61, this TF activated several key TFs and many SCW biosynthetic genes five- to ten-fold more strongly than EgrNAC26 by 14 hpt. These included TFs *BLH6*, *C3H14*, *KNAT7*, *MYB46*, *SND2* and *SND3*, cellulose-associated *CesA4*, *CesA7*, *CesA8* and *COBL4*, xylan-associated genes such as *IRX9*, *IRX10*, *IRX15*, *GUX1*, *GUS2* and *PARVUS*, and lignin-associated genes such as *CCoAOMT7*, *LAC4* and *LAC17* (Table 1). This, despite a lower transfection efficiency for the EgrNAC61-OX construct (Fig. S3b). In contrast, although PCD genes were expressed in all datasets, *BFN1*, *MC9*, *XCP1* and *XCP2* were upregulated by as much as an order of magnitude more in EgrNAC26-OX compared to EgrNAC61-OX at 14 h (Table 1). Supported by the apparently stronger transactivation ability of EgrNAC61 in yeast (demonstrated by its persistent growth at 15 mM 3AT, Fig. 1), our results demonstrate that EgrNAC61 is a more potent transcriptional activator of the Arabidopsis SCW pathway compared to EgrNAC26, while the latter shows a similar trend for PCD-associated genes in particular.

**Figure 4:**
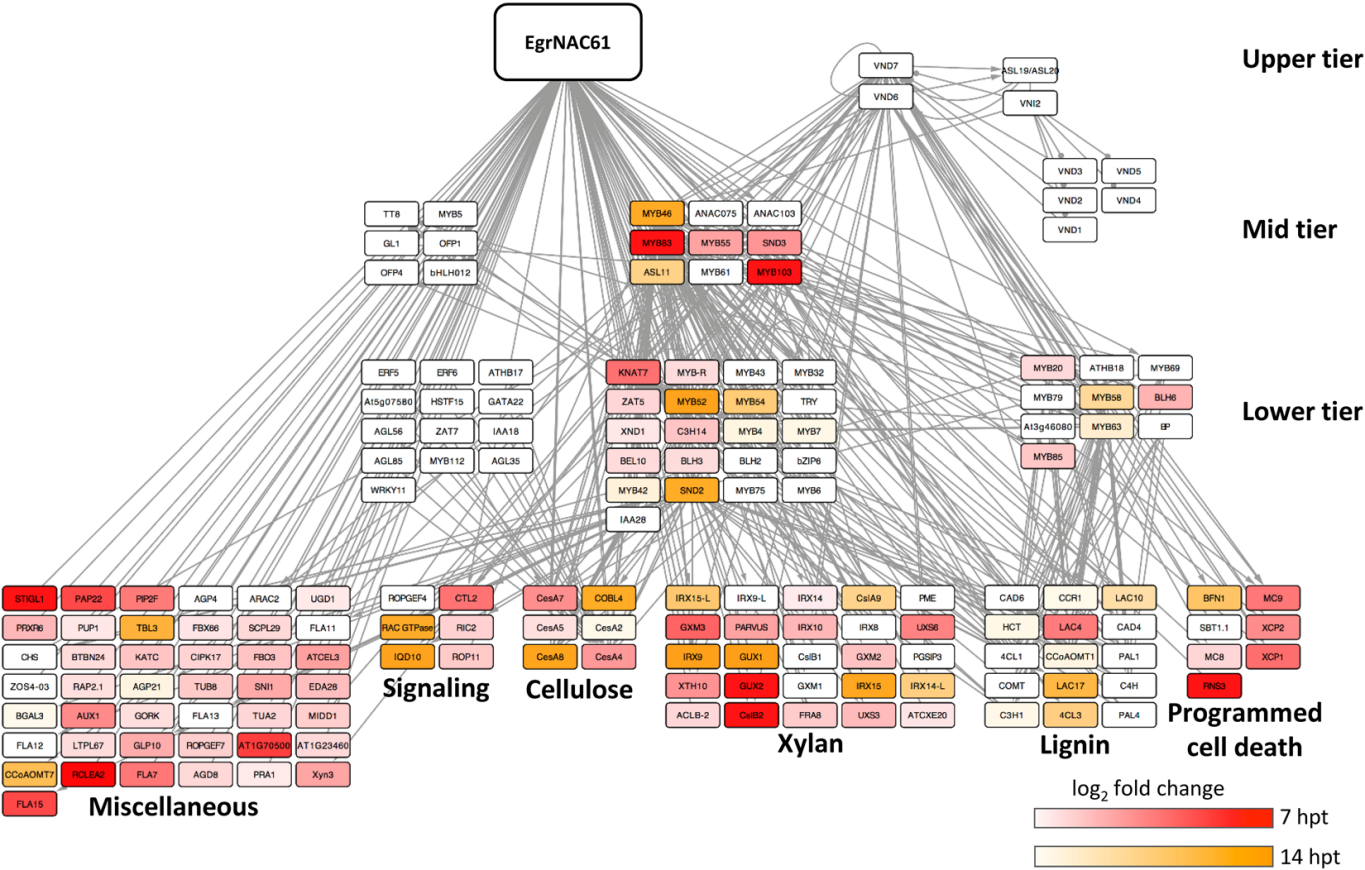
Time-course activation of the Arabidopsis SCW transcriptional network following *EgrNAC61* induction. The red heatmap indicates the fold change (log_2_) of transcripts activated at 7 hpt (hours post-transfection). The orange heatmap indicates the fold change (log_2_) of nodes that were exclusively upregulated at 14 hpt. Structural genes (bottom tier) are grouped according to biological function. The transcriptional network scaffold was adapted from Hussey *et al*.^52^.

**Figure 5:**
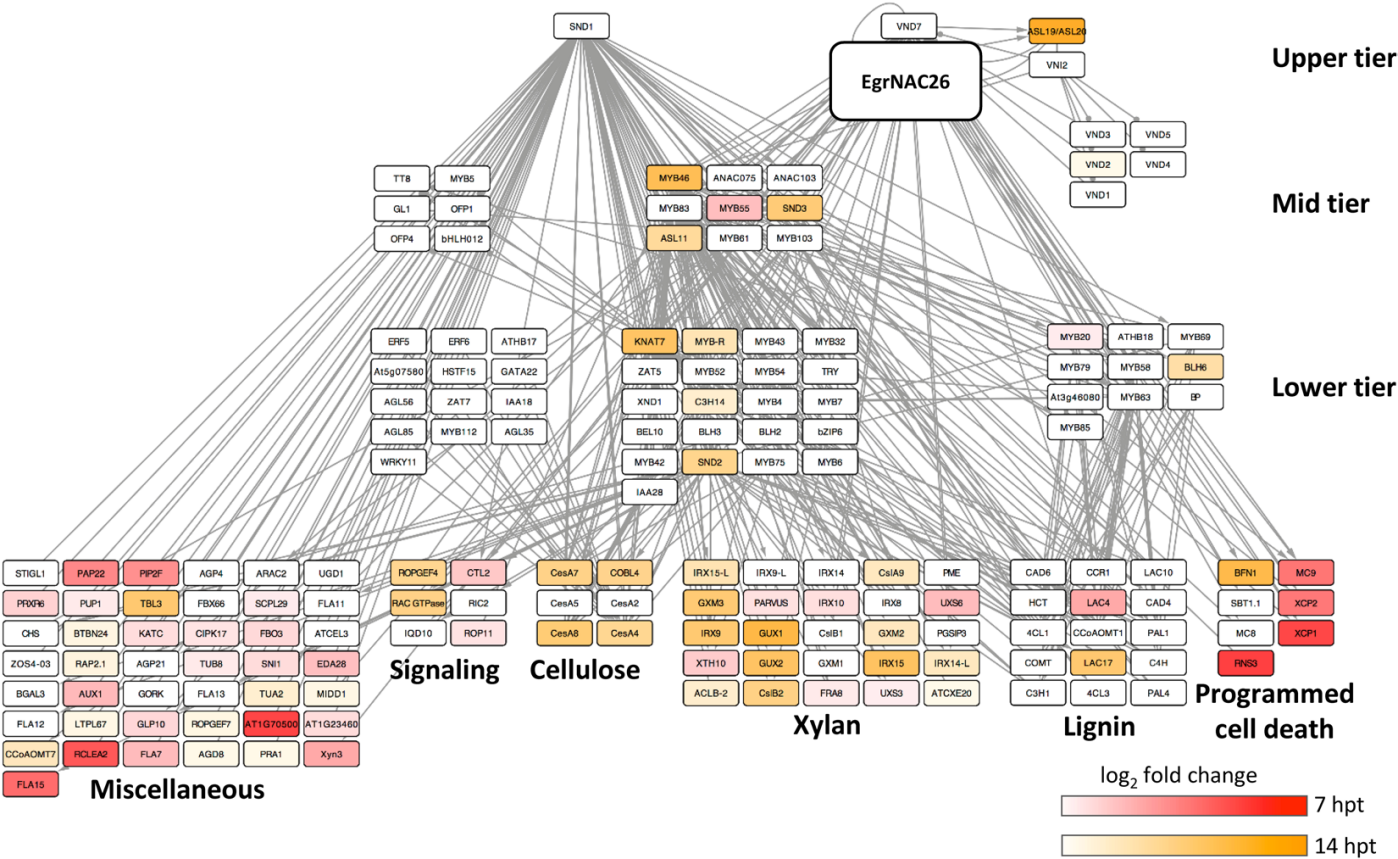
Time-course activation of the Arabidopsis SCW transcriptional network following *EgrNAC26* induction. The red heatmap indicates the fold change (log_2_) of transcripts activated at 7 hpt (hours post-transfection). The orange heatmap indicates the fold change (log_2_) of nodes that were exclusively upregulated at 14 hpt. Structural genes (bottom tier) are grouped according to biological function. The transcriptional network scaffold was adapted from Hussey *et al*.^52^.

To understand the temporal activation of SCW transcriptional network nodes following *EgrNAC26* and *EgrNAC61* induction in Arabidopsis protoplasts, we mapped the DEGs to a curated Arabidopsis SCW transcriptional network compiled from the literature^52^. Time-lapse activation of the known SCW transcriptional network nodes by EgrNAC61 and EgrNAC26 is represented in Figs 4 and 5, respectively. EgrNAC61 induced strong, early upregulation of second and third tier TFs as well as structural genes associated with cellulose, hemicellulose and lignin biosynthesis by 7 hpt and a few additional targets in all tiers by 14 hpt (Fig. 4). The effect of EgrNAC26 induction clearly differed from that of EgrNAC61, where strong activation of PCD-associated genes but weak activation of only a few SCW structural genes and second- or third-tier TFs were apparent at 7 hpt, with a better representation of mid-tier SCW-related TFs and biosynthetic genes by 14 hpt, although weakly activated compared to EgrNAC61-OX (Fig. 5). Given that *EgrNAC26* and *EgrNAC61* transfections were performed in parallel on a common preparation of protoplasts, these results demonstrate a weaker and possibly delayed onset of SCW structural gene activation for EgrNAC26 in comparison. Finally, we noted that two clusters of mid- and lower-tier TFs implicated in the SCW network^52^ were unaffected by induction of either EgrNAC26 or EgrNAC61 (Figs 4 and 5, left). These clusters are not known to be activated by SWNs, consistent with our results and suggesting an independent route of regulation.

## Discussion

In this study, we explored the temporal transactivation dynamics of Arabidopsis genes (Brassicales) by homologs of SCW master regulators of fibre and metaxylem vessel formation from *E*. *grandis*, a commercially important hardwood. Our results provide (1) insight into the possible roles of EgrNAC26 and EgrNAC61 SWNs in fibre and vessel SCW regulation in eucalypts, (2) evidence of considerable conservation in SWN gene targets between *Eucalyptus* as a representative of the basal eudicot lineage the Myrtales which divergence from the Brassicales over 100 million years ago^69^, and (3) a demonstration of the regulatory dynamics and specific targets of orthologous master regulators associated with fibre and vessel differentiation in a heterologous context typical of a synthetic biology strategy.

Few studies have explored the genome-wide gene targets of Arabidopsis SND1 and VND6/VND7^9,24,36,70^. These previous investigations revealed preferential activation of PCD-associated genes such as *XCP1* and *XCP2* during vessel differentiation by VND6 and VND7. In woody angiosperms, only one study has reported the genomic targets of a fibre-associated SCW master regulator, in this case a time-course experiment of poplar stem differentiating xylem protoplasts transfected with *Ptr-SND1-B1*, a homolog of *SND1* from *P*. *trichocarpa*^71^. Ours is the first study to probe the global transcriptional targets of *EgrNAC26* and *EgrNAC61*, albeit in a heterologous system (Arabidopsis). One advantage of our approach is that the transfections for each construct were performed in parallel from a single protoplast preparation, allowing for a direct comparison of the gene targets and temporal activation dynamics of each SWN candidate. Two limitations should be noted: first, mesophyll protoplasts may not reflect the accessible chromatin landscape or contain the same co-factors as xylem mother cells. However, SWNs and their homologs are known to be particularly powerful SCW master regulators, sufficient for ectopic tracheary element transdifferentiation in many cell types^20,72–74^. The inherent transcriptional transactivation properties of EgrNAC26 and especially EgrNAC61 in yeast (Fig. 1) and the extensive and significant enrichment of SCW-associated biological processes in all EgrNAC26-OX and EgrNAC61-OX DEG datasets (Fig. 3b) support this statement. Thus, we can infer that EgrNAC26 and EgrNAC61 activate homologs of genes similarly in protoplasts as they do in developing xylem. Second, since the use of a heterologous system may yield *Arabidopsis*-specific targets and cannot detect novel gene targets unique to *E*. *grandis*, validation of the *Eucalyptus* orthologs of the target genes in this study is recommended.

Given the predominant fibre- and vessel-preferential SCW regulatory functions of SND1 and VND6 respectively in Arabidopsis, we expected to observe similar cell type associations for their putative *E*. *grandis* orthologs EgrNAC61 and EgrNAC26. In contrast to this hypothesis, we observed a substantial overlap in promoter activity between *EgrNAC61pro* and *EgrNAC26pro* in during Arabidopsis vascular development, namely in seedling vessels (Fig. 2). The *EgrNAC26* promoter-driven reporter expression in Arabidopsis primary xylem vessels was substantially weaker, however. It is possible that the *EgrNAC26* promoter construct, which included only 1,223 bp upstream of the predicted transcription start site due to a large 2,422 bp 5′ UTR region containing an embedded intron, lacked a critical *cis-*element for stronger expression, or that the promoter is regulated differently in *E*. *grandis*.

In comparison with our promoter analysis, expression of the aspen *SND1* homolog peaked in phloem and early-onset SCW deposition in the xylem from cryosectioned xylogenesis developmental series^29^, consistent with our observation for developing xylem although it is unclear why no *EgrNAC61pro* activity was observed in phloem fibres (Fig. 2b). The aspen series^29^ did not include cork cambium, but we here report strong GUS induction by *EgrNAC61pro* in this tissue layer, while *EgrNAC26pro* was not assessed. Cork (phellem) consists of lignified and suberized cells that undergo PCD and have cell walls in which a guaiacyl-rich lignin backbone predominates^75,76^. Phloroglucinol staining revealed small amounts of lignin in the phellem of the poplar transgenics (Fig. 2b, bottom panel). Since *EgrNAC61* activates the phenylpropanoid pathway (Figs 3, 5), we postulate that it has been co-opted to activate phellem lignification in the cork cambium too.

In addition to its homology to VND6, several lines of evidence point to a vessel-specific SCW regulatory role for EgrNAC26 in the transient expression experiment: (i) EgrNAC26 transfection uniquely induced vessel-specific regulators of SCW deposition, among them *LATERAL ORGAN BOUNDARIES 30* (*LBD30/ASL19*), *VND2* and *TRACHEARY ELEMENT DIFFERENTION-RELATED 7* (*TED7*). *LBD30*/*ASL19* and *LBD18*/*ASL20* are targets of VND6 that participate in a positive feedback loop during vessel differentiation^77^, and VND2 is a vessel-specific SCW master regulator that interacts with VND7^15,23,74^. TED7 is a vessel-specific protein with an unknown role in tracheary element SCW formation, acting with TED6 and interacting with the SCW-related CesA complex^78^. While *TED6* was significantly upregulated by both EgrNAC26 and EgrNAC61 at 14 hpt (Table 1), the exclusive activation of *TED7* at the earliest time point by EgrNAC26 suggests a vessel-related specialization. (ii) EgrNAC26 activated proteins associated with SCW pit formation in vessels, uniquely among them *RHO GUANYL-NUCLEOTIDE EXCHANGE FACTOR 4* (*ROPGEF4*) and *MICROTUBULE-ASSOCIATED PROTEIN 70-5* (*MAP70-5*) (Table 1). ROPGEF4 is essential for pit formation through its highly localized activation of the small GTPase ROP11, which was a common target of EgrNAC26 and EgrNAC61 in addition to the microtubule-associated protein MIDD1 and the ROP11-deactivating protein ROPGAP3^79^. MAP70-5 is a tracheary element-specific microtubule-implicated protein, the abundance of which has a profound effect on the frequency of pitted, reticulated or smooth wall patterns observed in transdifferentiated cells^80^. Thus, its strong upregulation at 14 hpt in EgrNAC26-OX may re-structure the cytoskeleton for vessel-specific microtubule-directed SCW deposition. (iii) EgrNAC26 transfection induced stronger activation of the chief genes associated with autolysis of tracheary elements during PCD, namely *XCP1*, *XCP2* and *MC9*^46^, as well as *BIFUNCTIONAL NUCLEASE 1* (*BFN1*), the closest homolog of the *Zinnia* endonuclease *ZEN1* known to degrade nuclei in tracheary elements^41^ (Table 1). The exclusive enrichment of “programmed cell death” as a biological function term in the early induction (7 hpt) of *EgrNAC26* (Fig. 3b) strengthens this observation. A ribonuclease with a currently unknown role in xylem PCD, *RNS3*, was rapidly induced following transfection by EgrNAC26 and EgrNAC61 (Table 1), while expression of suspected but non-validated xylem PCD genes *XSP1* and *SBT1*.*1* were not affected in this experiment.

We also observed what appears to be a much weaker and possibly delayed response in SCW target activation by EgrNAC26 compared to EgrNAC61. Interestingly, the expression of *EgrNAC26* in protoplasts was substantially lower than that of *EgrNAC61*, despite the use of the same constitutive promoter and the fact that the transfection efficiency for *EgrNAC26* was somewhat higher (Figs S3b, S5a). We cannot currently explain this phenomenon. However, several promoter transactivation experiments in *Populus* and Arabidopsis showed that NST clade SWNs activate mid-level regulators *PtrMYB021* (i.e. *MYB46*), *PtrMYB020* (*MYB83*), *PtrMYB128* (*MYB103*), *PtrNAC156* (*SND2*), *PtrKNAT7* (*KNAT7*) and *PtrMYB28* (*MYB58/MYB63*) more strongly than VND clade SWNs^13,28,30,55^, a trend that also emerged in our time-course experiment where SCW structural genes were preferentially activated by EgrNAC61 much earlier and to a stronger level than for EgrNAC26 (Figs 4, 5). Since fibre cells exhibit thicker SCWs than vessels, stronger activation of SCW structural genes in fibres is developmentally plausible, and transcript levels of SCW cellulose, xylan and lignin genes appear higher in laser micro-dissected fibres compared to vessels in *Populus* in support of this^81^. An enrichment for primary metabolic processes in EgrNAC61-OX (Fig. 3b) may reflect a metabolic shift toward carbon investment in this carbon sink which is in agreement with previous observations of several amino acid pathways being upregulated during *in vitro* tracheary element transdifferentiation^82^. Fibre-specific mutant phenotypes for EgrNAC61-preferential targets such as *SND2* and *SND3*^13,83,84^ suggest that EgrNAC61 is an important fibre SCW regulator, but the clear reporter gene expression conferred by its promoter in Arabidopsis vessels and all cell types in the developing xylem in poplar plants (Fig. 2) suggests a dual role in SCW formation in fibres and vessels, and possible other xylem cells.

Given the combined evidence of vessel-specific specialization of EgrNAC26 and preferential PCD activation, versus vessel- and fibre-preferential expression of EgrNAC61 and strong activation of SCW structural genes and TFs following induction in Arabidopsis protoplasts, we favour the hypothesis that EgrNAC61 is the chief fibre SCW master regulator that plays a supporting role in vessel development, while EgrNAC26 complements EgrNAC61 in vessels with PCD and SCW pit-related regulatory functions. The potential role of *EgrNAC61* in regulating cell wall deposition in cork cells is an interesting topic for further investigation.

## Methods

### Construct preparation

Promoter fragments of 2,009 bp (*EgrNAC61* promoter; Eucgr. E01053), 3,660 bp (*EgrNAC26* promoter; Eucgr. A02887) and 1,115 bp (*VND6* promoter; AT5G62380) directly upstream of the translational start site were PCR-amplified from genomic DNA (Table S1), cloned into pCR8/GW/TOPO*®* (EgrNAC61) or pENTR/D-TOPO*®* (*EgrNAC26* and *VND6*) entry vectors (Invitrogen, Carlsbad, CA) and transferred to pBGWFS7^85^ using the Gateway LR ClonaseII Enzyme Mix (Invitrogen). For *Populus GUS* constructs, the promoters were transferred to pMDC162^86^. Developing secondary xylem RT-PCR products of *EgrNAC26* and *EgrNAC61* coding regions were cloned into pCR8/GW/TOPO*®* and transferred to the pUC-35S-Rfa-35S-GFP protoplast expression vector^71^ and pDEST-GBKT7 yeast expression vector^87^ similarly using LR recombination. Empty vector controls of the expression vectors were produced through LR recombination with a self-ligated pCR8/GW/TOPO® vector. Large-scale plasmid isolations for protoplast transfections were performed using the PureLink HiPure Plasmid FP Maxiprep Kit (Invitrogen), or caesium chloride purification^88^. The pHBT::sGFP(S65T)-NOS vector^89^ was used as a fluorescent marker to assess the effect of different plasmid purification methods on protoplast transfection efficiency.

### Plant transformation and GUS analysis

The pBGWFS7 constructs (*VND6pro::GFP:GUS*, *EgrNAC26pro::GFP:GUS* and *EgrNAC61pro::GFP:GUS*) were transformed into *Arabidopsis thaliana* using the *Agrobacterium*-mediated floral dip method^90^ and the transformed lines were selected on half-strength MS agar medium using Glufosinate-ammonium (20 mg/l). Transgenic Arabidopsis seeds were surface-sterilized, plated on nonselective nitrate-enriched media and allowed to germinate for 2–3 weeks. For poplar transformation, the *EgrNAC61pro::GUS* construct in the pMDC162 backbone was introduced into poplar hybrid *Populus alba* × *P*. *tremula* 717-1B4 by co-cultivation of *Agrobacterium* solution with leaf discs followed by shoot regeneration on selective media containing 30 mg/l Hygromycin. Transgenic poplar plants were propagated using greenwood stem cuttings and young developing stems (internodes 1 and 5) were harvested. GUS staining was performed as described by Spokevicius *et al*.^91^, excluding acetone treatment, but adding 0.5 mM potassium ferrocyanide and 0.5 mM potassium ferricyanide. After incubation at 37 °C overnight, seedlings were destained in 70% ethanol and visualized with differential interference contrast and brightfield microscopy.

### Yeast transactivation assays

The pDEST-GBKT7 constructs containing *EgrNAC61* or *EgrNAC26* were used to transform *Saccharomyces cerevisiae* strains AH109 and Y2HGold (Clontech™, CA, USA) through lithium acetate/polyethylene glycol-mediated transformation as previously described^92^. Transformed colonies were screened on media lacking tryptophan (Trp^-^), media lacking tryptophan and histidine (Trp^-^ His^-^), as well as Trp^-^ His^-^ media supplemented with 1 mM or 15 mM 3-amino-1,2,4-triazole (3AT, Sigma-Aldrich®, South Africa). Trp^-^ His^-^ media was prepared using -Trp-Leu-His drop-out supplement (Clontech™) and adding the appropriate amount of leucine supplement (Sigma-Aldrich®). Colonies were incubated for 3 days at 30 °C.

### Isolation and transfection of Arabidopsis leaf mesophyll protoplasts

*A*. *thaliana* ecotype Columbia (Col-0) seedlings were grown for four weeks at 22 **°**C under a 10 h photoperiod prior to mesophyll protoplast isolation from leaves as previously described^93^. Multiple plants were pooled for protoplast isolations and de-bulked to form three independent biological replicates. Large-scale transfections with pUC-35S-EgrNAC26-35S-GFP, pUC-35S-EgrNAC61-35S-GFP, and pUC-35S-NOS-35S-GFP (empty vector) constructs were conducted using 4 × 10^5^ protoplasts for each of three technical replicates per biological replicate (n = 3) to account for transfection variation and incubated for 7 h and 14 h at room temperature in the dark. Technical replicates were bulked and harvested through centrifugation at 500 × *g*. Of the harvested cells, 10 μl was used to determine transfection efficiency (at 14 hpt only) and the remaining cells were flash-frozen and stored at −80 **°**C.

### RNA-seq analysis

Total RNA was isolated using the Qiagen RNeasy Mini Kit (Sigma-Aldrich®, South Africa) and RNA quality was determined using an Experion instrument (Bio-Rad). Stranded 100 nt paired-end (PE100) mRNA sequencing was performed at Beijing Genomics Institute (BGI, Hong Kong) on the Illumina HiSeq4000 platform using fragment sizes of up to 200 bp. Raw RNA-seq reads passing initial quality filtering by BGI were further assessed for quality with FastQC^94^. The RNA-seq reads were formatted with FastQGroomer^95^ prior to mapping to the *A*. *thaliana* genome (TAIR10) using TopHat 2^96^. The mapped RNA-seq reads were assigned to gene models and the fragments per kilobase of transcript per million reads mapped (FPKM) values for each mapped transcript was calculated in Cufflinks^97^ using the *A*. *thaliana* TAIR10 annotation. The list of genes generated by Cufflinks was compiled into a single list with all nine libraries and Principle Component Analysis (PCA) was applied in R^98^ using the prcomp function^99^. Differentially expressed genes (DEG) were identified using the mapped RNA-seq reads in Cuffdiff^97^, with FPKM ≥1, false discovery rate (FDR) >0.05 and fold change ≥2 or ≤0.5.

The BiNGO plugin in Cytoscape^100,101^ was used to determine overrepresented Gene Ontology (GO) terms in the up- and down-regulated *EgrNAC26 and EgrNAC61* DEG datasets. A hypergeometric test with Benjamini and Hochberg FDR correction with a *P*-value cut-off of 0.05 was applied with the whole *A*. *thaliana* annotation as a reference set.

### Data availability

The RNA-seq data reported in this study is freely available at the NCBI Sequence Read Archive (https://www.ncbi.nlm.nih.gov/sra), accession SRP117192.

## Electronic supplementary material

Supplementary information

Dataset 1
