# Supplementary material for: Temporal analysis of Arabidopsis genes activated by Eucalyptus grandis NAC transcription factors associated with xylem fibre and vessel development
Source: Sci Rep. 2018 Jul 20;8:10983. doi: 10.1038/s41598-018-29278-w (PMC6054625; doi:10.1038/s41598-018-29278-w)
Supplement: Supplementary file 1 — Supplementary information [file 41598_2018_29278_MOESM1_ESM.pdf]

Temporal analysis of *Arabidopsis* genes activated by *Eucalyptus grandis* NAC transcription factors associated with xylem fibre and vessel development

Laubscher, M.[1], Brown K.[1], Tonfack, L.B.[2], Myburg, A.A.[1], Mizrachi, E.[1] and Hussey, S.G.[1*]

[1]Department of Genetics, Forestry and Agricultural Biotechnology Institute (FABI), Genomics Research Institute (GRI), University of Pretoria, Private Bag X28, Pretoria, South Africa 0002

[2]Plant Physiology and Improvement Unit, Laboratory of Biotechnology and Environment, Department of Plant Biology, University of Yaoundé I, P.O. Box 812, Yaoundé, Cameroon

*corresponding author email: steven.hussey@fabi.up.ac.za

Supplementary information

Supplementary Tables

Table S1. Primer information

Gene	Primer sequences	Amplicon size
EgrNAC26 CDS	5’ ATGAACACCTTTTCACGTGTC 3’	1047 bp
	5’ TCACTTCCAGAGTTCAATT 3’	
EgrNAC61 CDS	5’ ATGAACCTGTCCATAAACGGCCAGTC 3’	1200 bp
	5’ TTATACCGACAAATGACGTAATGGGTCAGA 3’	
VND6 promoter	5’ CACC-AAGTGGTTAACTTTCAACAATG 3’	1115 bp
	5’ GCGAGACTTTCgATTTGATCTTT 3’	
EgrNAC26 promoter	5’ CACC-TTAAATCATACAACCGACGAATTG 3’	3660 bp
	5’ AAGGTGTTGATATTCgATCCTCTTTAG 3’	
EgrNAC61 promoter	5’ CATGTGTGCGTTTGAGAGAGAG 3’	2009 bp
	5’ CAGGTTCATTTTCTCTTACAACAG 3’	

Table S2. Stranded RNA-seq mapping efficiency.

Sample	Aligned read pairs	Multiple alignments	Concordant pair alignment rate (%)
EV rep 1 (7 hpt)	24,182,050	1,486,104	90.3
EV rep 2 (7 hpt)	21,721,354	1,482,544	92.5
EV rep 3 (7 hpt)	24,081,533	1,723,656	91.6
EgrNAC61 rep 1 (7 hpt)	17,376,444	1,269,280	74.1
EgrNAC61 rep 2 (7 hpt)	22,571,304	1,616,015	92.7
EgrNAC61 rep 3 (7 hpt)	21,806,637	1,450,532	91.0
EgrNAC26 rep 1 (7 hpt)	24,350,861	1,428,861	93.4
EgrNAC26 rep 2 (7 hpt)	22,637,260	1,651,617	91.8
EgrNAC26 rep 3 (7 hpt)	17,711,690	1,193,430	91.9
EV rep 1 (14 hpt)	34,878,470	2,267,995	93.4
EV rep 2 (14 hpt)	31,206,902	2,298,962	91.4
EV rep 3 (14 hpt)	35,066,978	3,037,974	93.0
EgrNAC61 rep 1 (14 hpt)	36,392,435	3,246,140	92.7
EgrNAC61 rep 2 (14 hpt)	35,068,051	2,926,106	92.9
EgrNAC61 rep 3 (14 hpt)	33,429,285	2,555,963	92.8
EgrNAC26 rep 1 (14 hpt)	33,604,065	2,476,613	91.6
EgrNAC26 rep 2 (14 hpt)	29,878,674	4,127,714	91.1
EgrNAC26 rep 3 (14 hpt)	32,980,097	2,553,151	92.4

EV, empty vector control
hpt, hours post-transfection

Table S3. Transcription factor genes induced by EgrNAC61 and EgrNAC26. The fold change for each gene is shown for the EgrNAC26-OX and EgrNAC61-OX overexpression datasets. Transcription factors were identified as those annotated with the molecular function "sequence-specific DNA binding" (GO:0003700).

			Fold change			
			EgrNAC26-OX		EgrNAC61-OX	
**Family**	**Gene ID**	**Gene name**	**7 hpt**	**14 hpt**	**7 hpt**	**14 hpt**
AP2	AT5G65510	AIL7	-	-	-	0.4
B3	AT4G32010	HSI2-L1	-	-	-	2.3
	AT2G36080	ABS2	-	-	2.2	-
bHLH	AT5G65640	bHLH093	-	-	-	0.5
	AT5G43650	BHLH92	-	2.3	-	2.9
	AT1G26260	CIB5	3.2	2.8	3.3	2.3
	AT2G20180	PIF1	-	-	-	0.5
	AT2G43010	PIF4	-	-	2.7	-
	AT3G07340		-	-	-	5.6
	AT4G25410		-	-	-	4.5
	AT2G22750		-	-	-	0.5
	AT4G01460		-	-	5.8	-
	AT5G65320		-	-	0.4	-
	AT2G46810		-	-	0.3	-
bZIP	AT1G68880	bZIP8	2.4	2.9	8.6	2.1
	AT2G41070	bZIP12	-	-	-	2.1
	AT3G51960	bZIP24	-	-	-	0.4
	AT5G06839	bZIP65	-	-	0.4	0.4
C2H2	AT4G35280	DAZ2	-	-	-	Inf*
	AT3G58070	GIS	-	-	43.9	-
	AT3G13810	IDD11	-	-	-	0.5
	AT2G41940	ZFP8	-	-	0.5	-
	AT5G03510		23.5	88.9	189.4	256.3
	AT4G27240		2.2	3.0	6.8	11.9
	AT1G02030		-	-	3.3	3.5
	AT2G28200		-	-	4.2	3.2
	AT3G60580		3.5	3.0	6.0	2.1
	AT5G60470		-	-	-	0.5
C3H	AT1G68200		-	-	5.0	5.6
	AT5G44260	TZF5	-	-	2.1	-
CO-like	AT1G73870	BBX16	-	-	-	2.0
DBB	AT1G75540	BBX21	-	-	2.2	-
Dof	AT5G60200	TMO6	-	-	2.8	3.4
ERF	AT4G25470	CBF2	-	-	-	0.5
	AT4G27950	CRF4	-	-	0.5	-
	AT1G21910	DREB26	-	-	-	0.3
	AT1G53170	ERF-8	-	-	2.5	-
	AT5G44210	ERF-9	-	4.5	2.7	15.7
	AT5G25190	ESE3	3.5	3.7	6.5	4.8
	AT1G06160	ORA59	-	-	-	0.3
	AT1G46768	RAP2.1	-	3.2	3.1	6.1
	AT1G15360	SHN1	-	-	-	Inf*
	AT5G07310		3.6	7.2	3.1	6.8
	AT5G21960		-	-	-	0.5
	AT2G44940		-	-	0.5	0.4
	AT5G67000		-	-	0.5	0.4
	AT1G33760		-	0.4	-	0.2
	AT1G77640		-	-	-	0.1
	AT5G51190		5.6	3.0	5.8	-
	AT4G32800		-	-	2.9	-
G2-like	AT3G10760		-	-	-	2.4
	AT2G02060		-	-	-	0.5
	AT2G40260		-	-	-	0.5
	AT1G49560		-	-	2.6	-

GATA	AT3G51080	GATA6	-	-	19.2	38.7
	AT4G32890	GATA9	-	33.4	-	45.7
	AT1G08010	GATA11	-	-	-	2.3
	AT5G56860	GATA21	-	-	3.5	-
GRAS	AT3G60630	HAM2	-	-	2.1	-
	AT5G17490	RGL3	-	-	-	2.0
HD-ZIP	AT4G16780	ATHB2	-	-	-	0.4
	AT1G69780	ATHB13	-	-	-	0.5
	AT2G34710	ATHB14	-	-	2.2	2.6
HSF	AT5G62020	HSFB2A	-	-	-	2.1
	AT2G41690	HSFB3	-	6.4	25.2	43.5
MIKC-MADS	AT2G03710	AGL3	-	-	3.5	2.4
	AT5G20240	PI	-	-	-	21.2
M-type MADS	AT1G22590	AGL87	-	-	2.2	-
MYB	AT1G22640	MYB3	-	-	-	2.4
	AT4G38620	MYB4	-	-	-	2.7
	AT2G16720	MYB7	-	-	-	2.2
	AT1G66230	MYB20	2.2	-	3.8	2.4
	AT5G40350	MYB24	-	-	-	2.2
	AT2G39880	MYB25	-	-	-	11.0
	AT5G23000	MYB37	-	-	-	0.5
	AT4G12350	MYB42	-	-	-	3.5
	AT3G48920	MYB45	-	-	-	0.4
	AT5G12870	MYB46	-	255.9	-	1161.7
	AT1G18710	MYB47	-	-	-	0.4
	AT5G54230	MYB49	-	-	-	3.4
	AT1G17950	MYB52	-	-	-	Inf*
	AT5G65230	MYB53	-	-	-	0.4
	AT1G73410	MYB54	-	-	-	84.8
	AT4G01680	MYB55	9.3	3.8	22.6	4.9
	AT1G16490	MYB58	-	-	-	28.3
	AT1G79180	MYB63	-	-	-	5.2
	AT2G23290	MYB70	-	-	2.2	-
	AT3G08500	MYB83	-	-	Inf*	Inf*
	AT4G22680	MYB85	-	-	6.9	6.8
	AT2G32460	MYB101	-	-	-	0.4
	AT1G63910	MYB103	-	-	Inf*	-
	AT1G74080	MYB122	-	-	0.5	0.4
	AT2G38090		-	12.3	3.5	48.4
	AT1G49010		3.2	4.6	10.9	6.1
MYB-related	AT3G10590		13.4	290.9	21.2	131.4
	AT1G18330	EPR1	-	-	0.4	-
NAC	AT4G28500	SND2	-	42.4	-	1085.8
	AT1G28470	SND3	-	88.1	33.4	416.9
	AT1G02220	ANAC003	-	-	-	0.4
	AT1G02230	ANAC004	-	0.5	-	0.4
	AT1G52880	ANAC018	-	-	-	0.5
	AT1G56010	ANAC021	-	-	-	0.5
	AT1G69490	ANAC029	3.3	2.3	11.9	3.6
	AT2G27300	ANAC040	-	15.2	-	13.9
	AT2G33480	ANAC041	2.7	3.4	7.6	5.8
	AT3G04070	ANAC047	-	-	-	0.4
	AT3G04420	ANAC048	-	0.5	-	0.4
	AT3G18400	ANAC058	-	-	Inf*	Inf*
	AT5G64530	ANAC104	-	-	2.5	-
NF-YA	AT1G54160	NF-YA5	-	-	3.0	6.1
Nin-like	AT4G35590	RKD5	-	-	0.4	-
RAV	AT3G25730	EDF3	-	-	-	2.6
SBP	AT1G53160	FTM6	-	-	-	Inf*
	AT5G18830	SPL7	-	-	-	2.1
TALE	AT1G75410	BLH3	-	-	3.4	4.4
	AT4G34610	BLH6	-	18.5	12.9	361.7
	AT1G19700	BEL10	-	-	3.5	5.7

	AT1G62990	IXR11	-	162.4	108.8	1139.7
TCP	AT3G15030	MEE35	-	-	-	3.3
	AT1G69690	TCP15	-	-	-	0.5
	AT5G08070	TCP17	-	0.5	-	0.5
Trihelix	AT1G33240	GTL1	-	-	2.5	-
WOX	AT4G35550	WOX13	2.1	2.7	2.3	2.7
WRKY	AT1G68150	WRKY9	-	-	0.4	0.4
	AT4G31800	WRKY18	-	-	-	2.4
	AT2G46400	WRKY46	-	-	-	0.4
	AT5G64810	WRKY51	-	-	-	0.5
	AT1G18860	WRKY61	-	-	-	0.4
	AT1G29280	WRKY65	-	-	0.5	0.5
	AT3G56400	WRKY70	-	-	-	0.5
	AT5G15130	WRKY72	-	0.4	0.5	0.4
	AT1G66600	ABO3	-	-	0.5	-
YABBY	AT1G08465	YAB2	-	-	-	2.2
ZF-HD	AT5G15210	ATHB30	-	-	2.4	2.8
Other	AT5G43700	AUX2-11	3.0	12.9	8.7	12.2
	AT1G15580	AUX2-27	-	4.5	7.4	5.7
	AT2G43060	IBH1	3.3	4.5	7.4	8.7
	AT2G21650	ATRL2	-	-	-	0.4
	AT1G43640	TLP5	-	-	-	19.3
	AT3G23050	AXR2	-	4.9	-	4.1
	AT4G14560	AXR5	-	4.0	-	2.9
	AT2G23380	CLF	-	-	-	3.3
	AT3G23030	IAA2	-	2.7	2.4	3.0
	AT1G04100	IAA10	-	-	2.1	2.7
	AT4G28640	IAA11	5.4	2.9	4.5	-
	AT3G16500	IAA26	-	-	-	0.5
	AT3G62100	IAA30	-	19.0	-	85.2
	AT5G57420	IAA33	-	-	-	Inf*
	AT4G20400	JMJ14	-	-	-	2.3
	AT5G04840		2.8	3.9	6.2	5.7
	AT1G11950		-	-	2.1	-

*Inf refers to instances where the FPKM was 0 in the empty-vector control, but was larger than 1 in the test sample.

hpt, hours post transfection.

Supplementary Figures

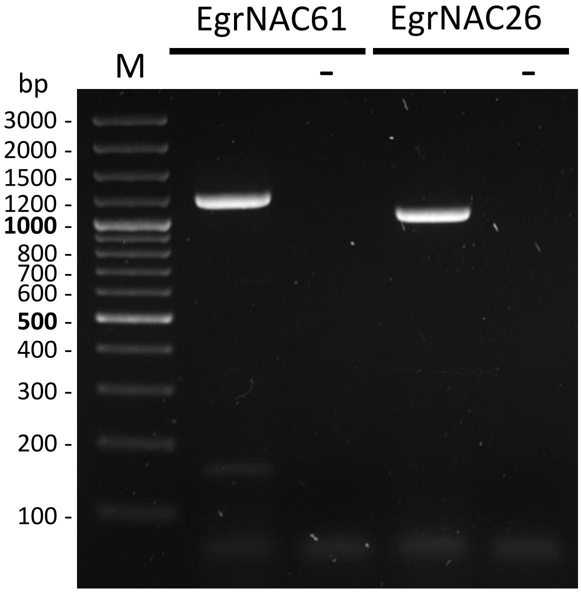

Figure S1. PCR amplification of *EgrNAC61* and *EgrNAC26* coding sequences from *E. grandis* developing secondary xylem cDNA. M, GeneRuler 100 bp DNA ladder PLUS; lanes indicated with (-) represent the template-free negative control. Expected amplicon sizes are 1,200 bp and 1,047 bp for *EgrNAC61* and *EgrNAC26*, respectively.

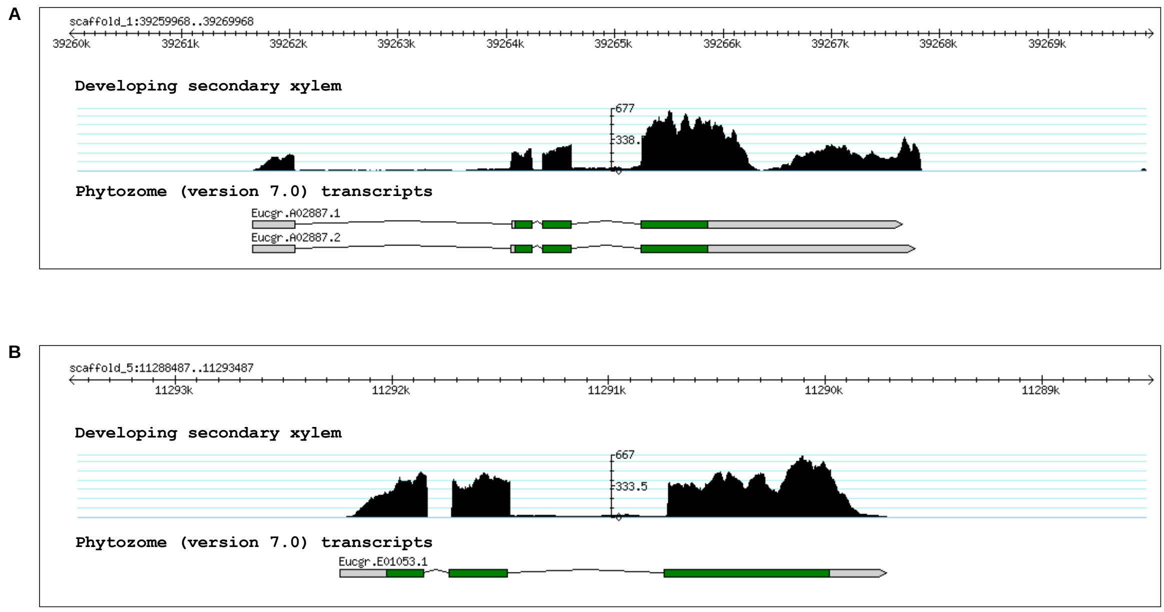

Figure S2. *E. grandis* RNA-seq expression data for EgrNAC26 (A) and EgrNAC61 (B). Exons are indicated in green, untranslated regions in grey, and spliced introns as a linker. Mapped RNA-seq read coverage in developing secondary xylem bulked across three individual samples is indicated in black. The data was visualized using the Gbrowse tool in EucGenIE[1], and adapted.

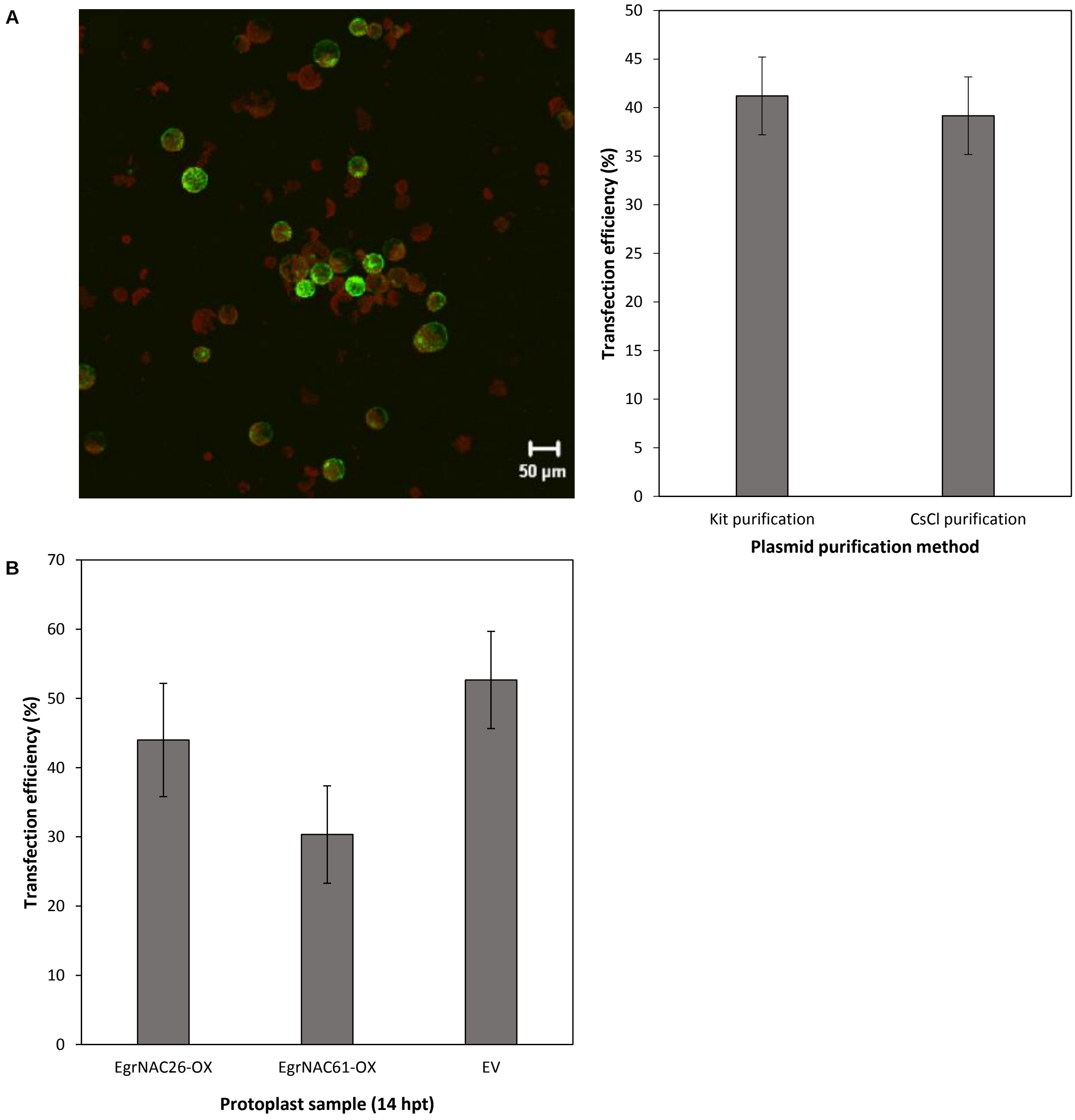

Figure S3. Arabidopsis mesophyll protoplast transfection efficiencies. (A) Analysis of plasmid purification method and transfection efficiency, determined with pHBT::sGFP(S65T)-NOS. Error bars indicate the standard error of ten independent transfections for each separate plasmid purification method. A representative confocal micrograph is shown on left, with GFP marker fluorescence (green) at 488 nm and chlorophyll autofluorescence (red) at 560 nm. Kit-based plasmid purification was used for all transfection experiments. (B) Transfection efficiencies of samples transfected with EgrNAC26-OX and EgrNAC61-OX constructs, at 14 hpt (hours post transfection). Error bars indicate standard deviation of three biological replicates. EV, empty vector control.

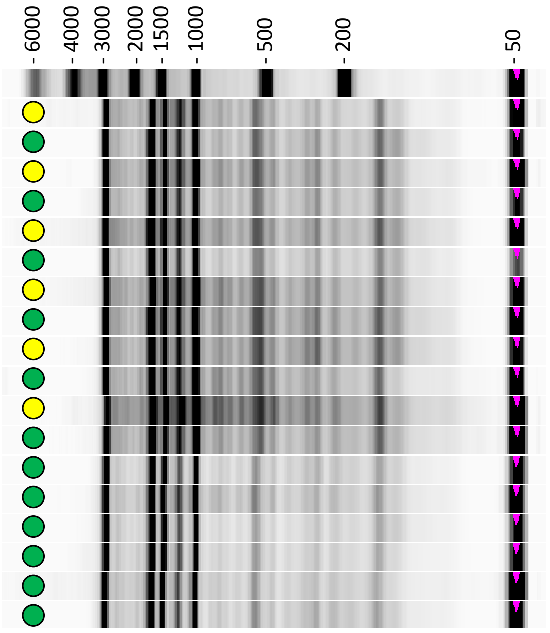

Well ID	Sample Name	RNA Concentration (ng/µl)	Ratio [28S/18S]	RQI
L	Ladder	160		
1	EgrNAC61-1 7h	154.03	0.89	6.7
2	EgrNAC26-1 7h	228.22	1.07	7.5
3	EV-1 7h	130.19	0.96	6.8
4	EgrNAC61-2 7h	249.82	1.16	7.6
5	EgrNAC26-2 7h	179.6	0.91	6.7
6	EV-2 7h	317.06	1.17	7.7
7	EgrNAC61-3 7h	208.33	0.93	6.7
8	EgrNAC26-3 7h	192.01	1	7.1
9	EV-3 7h	235.47	0.99	6.9
10	EgrNAC61-1 14h	166.55	1.03	7.4
11	EgrNAC26-1 14h	127.09	0.76	6.1
12	EV-1 14h	86.36	1.04	7.5
13	EgrNAC61-2 14h	218.86	1.29	7.8
14	EgrNAC26-2 14h	214.84	1.11	7.6
15	EV-2 14h	244.77	1.28	7.9
16	EgrNAC61-3 14h	225.39	1.19	7.9
17	EgrNAC26-3 14h	117.54	1.25	7.8
18	EV-3 14h	175.08	1.21	7.8

Figure S4. Experion analysis of RNA quality. Identities of the samples in each well of the pseudogel (left) are shown in the adjacent table. Samples are colour-coded according to RNA Quality Index (RQI) from green (RQI > 7.0) to yellow (6.0 < RQI < 6.9). EV, empty vector control.

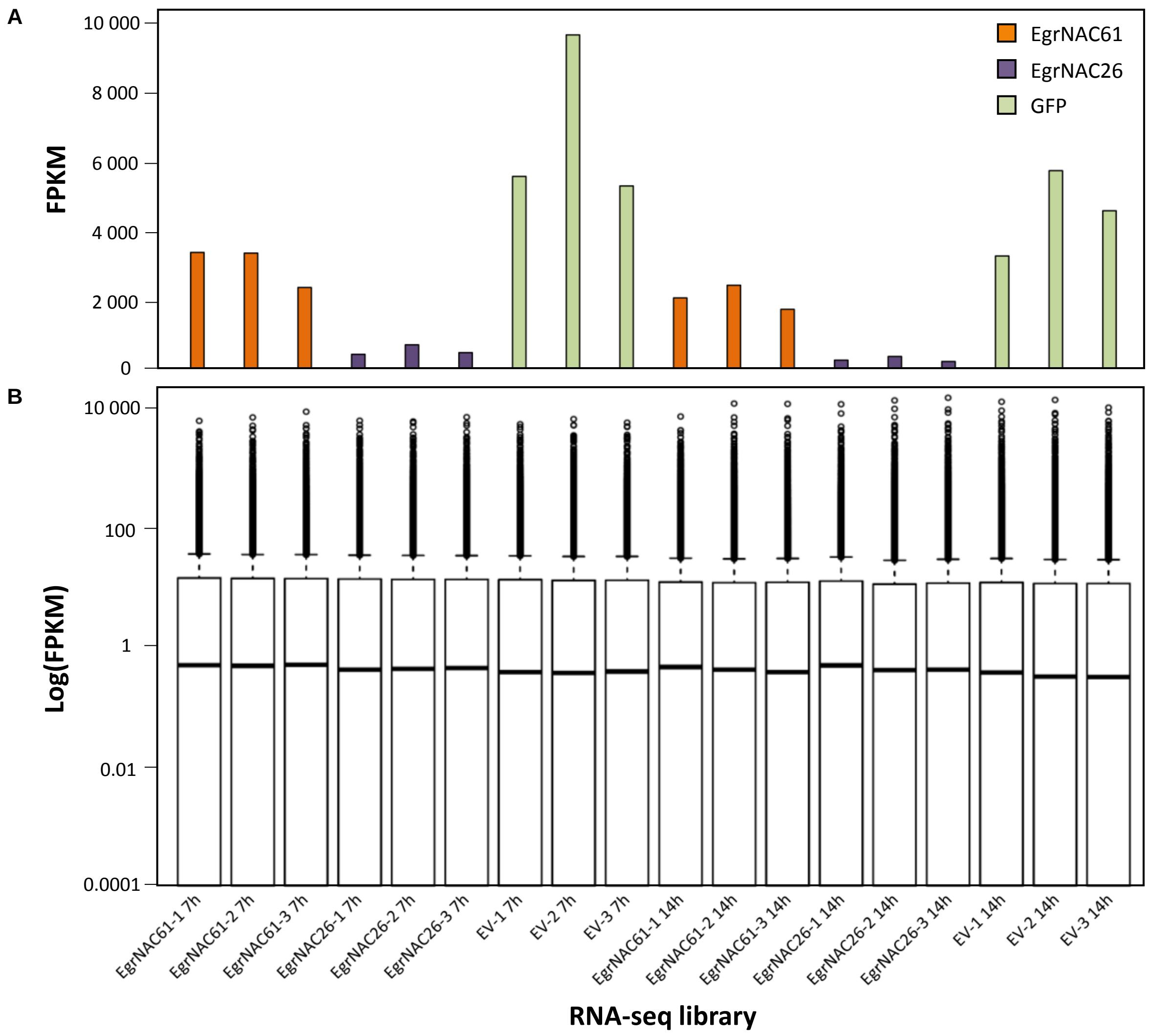

Figure S5. Transgene expression and transcript abundance distribution in transfected Arabidopsis leaf mesophyll protoplasts. (A) Expression levels of *EgrNAC61* (orange), *EgrNAC26* (violet) or *GFP* (green) transcripts for protoplasts transfected with *EgrNAC61*, *EgrNAC26* or an empty vector (EV) control constructs, respectively. (B) Boxplot of the FPKM values of the expressed genes (FPKM > 0) in the RNA-seq libraries.

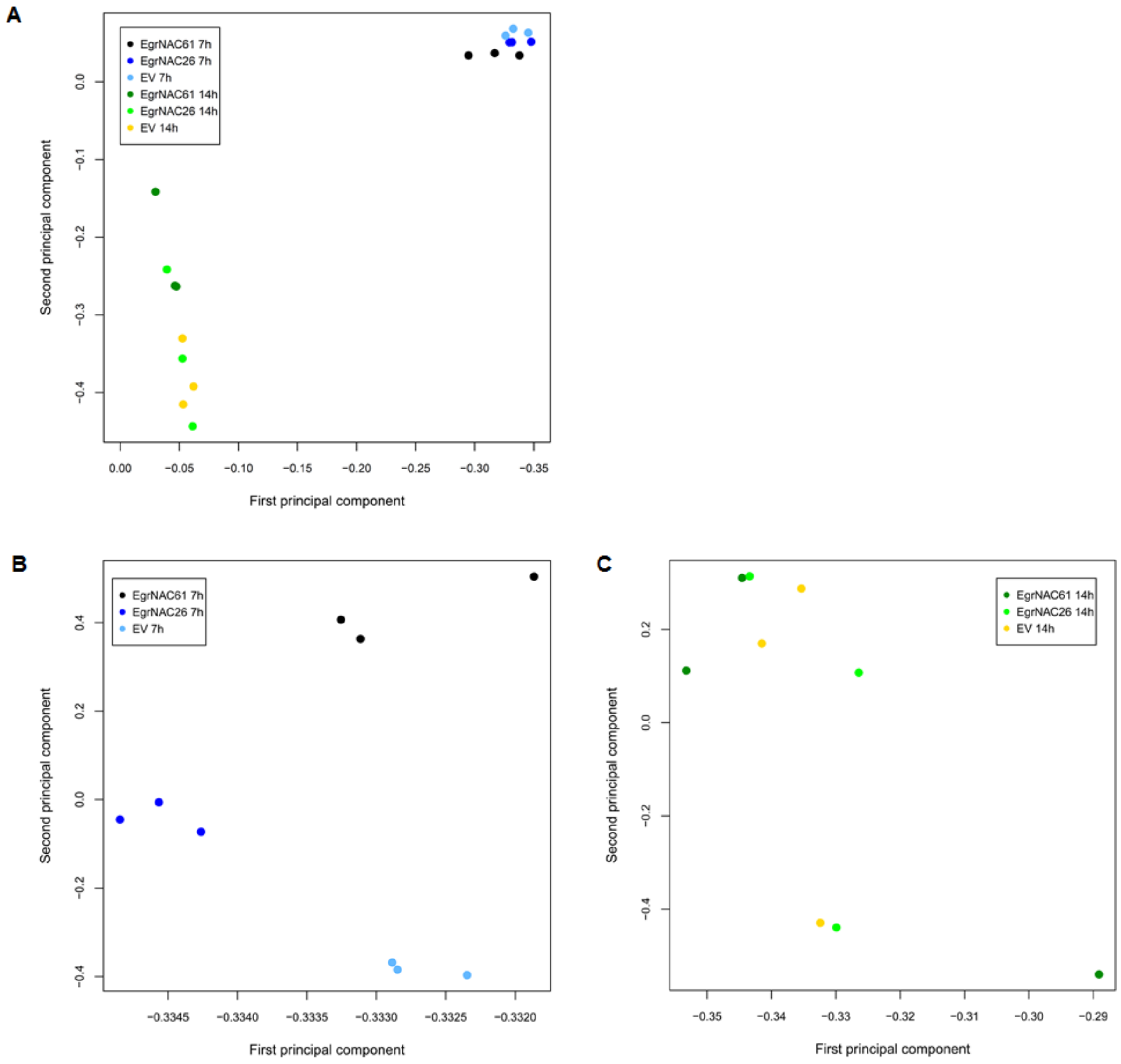

Figure S6. Principal component analysis of FPKM values in RNA-seq libraries. (A) All libraries, (B) 7 hours post-transfection, (C) 14 hours post-transfection. EV, Empty Vector Control.

A

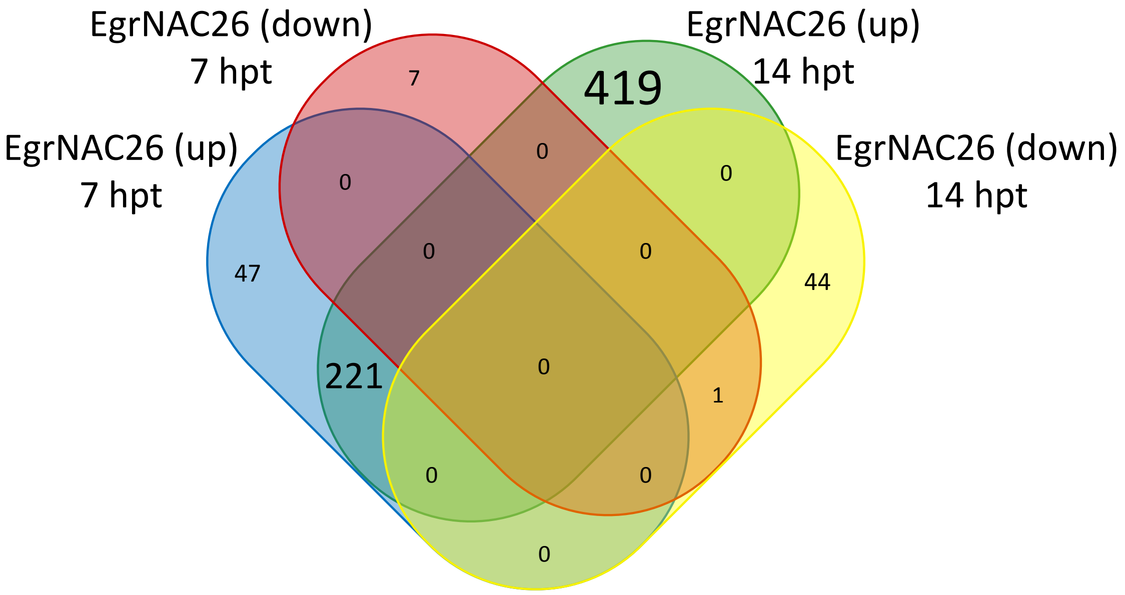

B

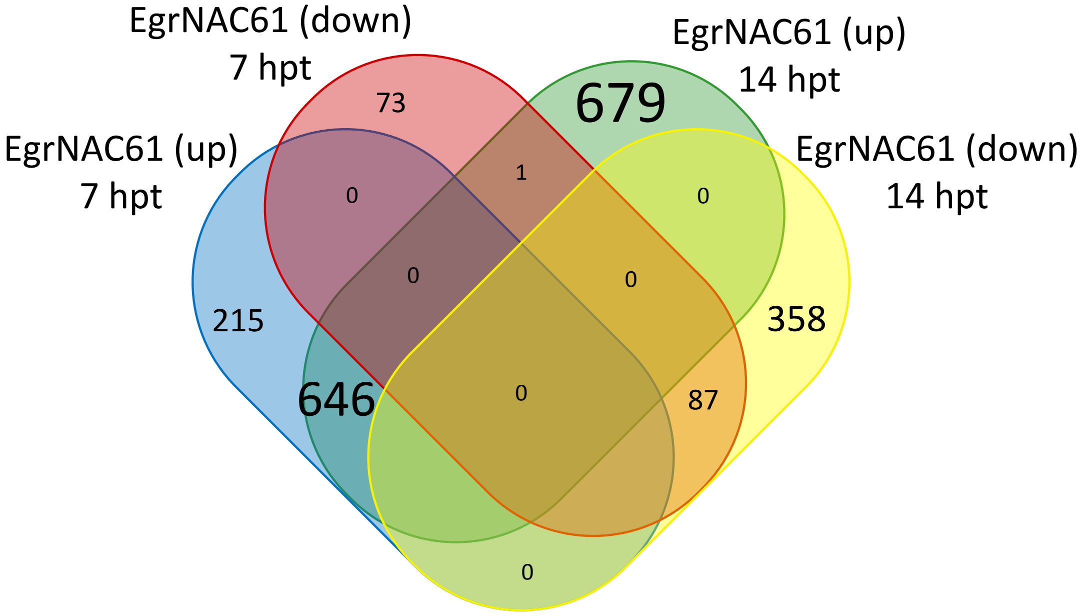

Figure S7. Venn diagrams of common and unique DEGs between 7 hpt and 14 hpt. (A) EgrNAC26-OX, (B) EgrNAC61-OX.

References

1 Hefer, C., Mizrachi, E., Joubert, F. & Myburg, A. The *Eucalyptus* genome integrative explorer (EucGenIE): a resource for *Eucalyptus* genomics and transcriptomics. *BMC Proceedings* **5**, O49 (2011).
